# Adaptive immunity to rhinoviruses: sex and age matter

**DOI:** 10.1186/1465-9921-11-184

**Published:** 2010-12-31

**Authors:** Melanie L Carroll, Stephanie T Yerkovich, Antonia L Pritchard, Janet M Davies, John W Upham

**Affiliations:** 1 School of Medicine, The University of Queensland, Brisbane, Australia; 2 Department of Respiratory Medicine, Princess Alexandra Hospital, Brisbane, Australia; 3 Queensland Centre for Pulmonary Transplantation and Vascular Disease, The Prince Charles Hospital, Brisbane, Australia

## Abstract

**Background:**

Rhinoviruses (RV) are key triggers in acute asthma exacerbations. Previous studies suggest that men suffer from infectious diseases more frequently and with greater severity than women. Additionally, the immune response to most infections and vaccinations decreases with age. Most immune function studies do not account for such differences, therefore the aim of this study was to determine if the immune response to rhinovirus varies with sex or age.

**Methods:**

Blood mononuclear cells were isolated from 63 healthy individuals and grouped by sex and age (≤50 years old and ≥52 years old). Cells were cultured with rhinovirus 16 at a multiplicity of infection of 1. The chemokine IP-10 was measured at 24 h as an index of innate immunity while IFNγ and IL-13 were measured at 5 days as an index of adaptive immunity.

**Results:**

Rhinovirus induced IFNγ and IL-13 was significantly higher in ≤50 year old women than in age matched men (p < 0.02 and p < 0.05) and ≥52 year old women (p < 0.02 and p > 0.005). There was no sex or age based difference in rhinovirus induced IP-10 expression. Both IFNγ and IL-13 were negatively correlated with age in women but not in men.

**Conclusions:**

This study suggests that pre-menopausal women have a stronger adaptive immune response to rhinovirus infection than men and older people, though the mechanisms responsible for these differences remain to be determined. Our findings highlight the importance of gender and age balance in clinical studies and in the development of new treatments and vaccines.

## Background

Rhinovirus (RV) infections are a key trigger for acute exacerbations of asthma in children and adults [1-4]. There is increasing evidence that this is linked to a subtle impairment of anti-viral immunity to RV and possibly other viruses. The nature of this impairment is ambiguous with some studies reporting airway epithelial cells and alveolar macrophages from asthmatics secrete less type I and type III interferon (IFN) than healthy people [5,6], while other investigators dispute this finding [7,8]. Altered innate immunity in asthma is not restricted to resident cells within the lung, but also seems to involve circulating leukocytes. Peripheral blood mononuclear cells (PBMC) from asthmatic children and adults show reduced IFNα secretion following *in vitro *exposure to viruses [9,10], and we have recently demonstrated reduced TLR7 function in PBMC from adolescents with asthma [11].

Adaptive immunity is also important for host defence against RV infection. High titres of RV specific antibodies are associated with protection from infection [12] as is the capacity of T-cells to secrete IFNγ after RV stimulation *in vitro *[13,14]. In asthma, the PBMC response to RV is characterized by deficient Th1 immunity [13-16], with reduced IFNγ secretion being associated with greater viral shedding after experimental RV infection [13,14].

Given that RV infections are so common, there is surprisingly little known about immunity to RV in healthy individuals; in order to fully elucidate whether there are perturbations in response in asthmatics, further understanding of this issue is vital. It is clear that a variety of infections affect men more often and more severely than women and that women generally make stronger immune responses to infections and vaccines compared to men, as reviewed by Klein *et al *and Fish *et al *[17,18]. This could also be seen utilising an *ex vivo *system examining T_H_2 polarised cultures, where women displayed a stronger regulation of type 2 T cells than men [19]. The explanation for these sex based differences may involve hormonal and genetic factors: oestrogens, progesterone and testosterone can all modulate many aspects of immune function [17,18], while two key genes involved in detecting viral RNA (*TLR7 *and *TLR8*) are located on the X chromosome [20]. Previous studies have also indicated that immune responses to viruses decrease with age [21].

There is little information in the literature on whether immune responses to RV vary in relation to sex and age. This is an important and relevant issue for research into respiratory diseases such as asthma where there is a female predominance in adult life. The aim of the current study was therefore to investigate markers of both innate and adaptive immune responses to RV in peripheral blood mononuclear cells (PBMC) from healthy men and women. The study was performed in pre- and post-menopausal females as well as age-matched males.

## Methods

### Patients

We recruited healthy adult volunteers with no history of asthma or other lung diseases. Females (n = 32) and males (n = 31) were divided into those aged up to 50 years, and those aged 52 years and older, as the median age of menopause in Australia is 51 yrs [22]. The women ≥52 years were not undertaking any hormonal therapy. One woman in the ≤50 year old group was currently taking the oral contraceptive pill. The subjects of the study had skin prick testing (SPT) to a panel of common allergens and were questioned about symptoms of allergy and lung diseases. The study was approved by the Princess Alexandra Hospital and the University of Queensland Human Research Ethics Committees, and informed consent was obtained from each subject.

### Rhinovirus generation and titration

Ohio HeLa cells and the major group RV strain RV16 were kindly donated by Professor Phil Bardin, Monash Medical Research Centre, Melbourne, Australia. RV stocks were generated by passage in Ohio HeLa cells as described previously [23] followed by purification over a sucrose gradient [24]. To determine the optimal concentration of RV, Ohio HeLa cells were seeded into a 96 well plate at a density of 1 × 10^4 ^cells per well in 150 μl RPMI containing 2% low endotoxin fetal bovine serum (FBS; Bovogen Biologicals, Victoria) and allowed to adhere overnight. The following day 10-fold serial dilutions of virus was added in triplicate to the cells at 50 ul/well and cultured at 37°C for 6 days. The media was removed and cells stained with 0.1% crystal violet solution in PBS to determine the amount of virus required to infect 50% of cells (TCID_50_).

### Cell separation and culture

PBMC were isolated from heparinised blood by density gradient centrifugation as previously described [11], and cultured at 1 × 10^6 ^PBMC in 24 well culture plates at a final concentration of 2 × 10^6 ^cells per/ml together with RV16 at a multiplicity of infection (MOI) of one; i.e. one virion per cell. Control cultures contained medium alone. RPMI media was supplemented with antibiotics, 2-mercaptoethanol and either 10% foetal calf serum (FCS; innate immune studies) or 5% autologous plasma (adaptive immune studies). Extensive comparative experiments showed that both FCS and autologous plasma supplemented media induced identical innate immune responses, though autologous plasma was preferred for adaptive immune studies in order to minimise foreign antigen exposure and because autologous plasma supplemented media was associated with consistently higher adaptive IFNγ synthesis (*data not shown*). The experiments that required depletion of antigen experienced/memory T cells from PBMC were performed using CD45R0+ immuno-magnetic beads (Miltenyi Biotech), according to the manufacturer's directions. Cultures were incubated at 37°C with 5% CO_2 _and supernatant was harvested for cytokine quantification by ELISA. Cell pellets were stored in 'RNA-protect' (Qiagen) until RNA was extracted. Initial time course experiments indicated that optimal expression of mRNA for IFN-stimulated genes and Th1-polarising genes was at 6 hours post-stimulation. The optimal time point for IFN-gamma-inducible protein 10 (IP-10, also known as CXCL10) secretion was at 24 hours post-stimulation while the optimal time point for the adaptive cytokines IFNγ and IL-13 was at 5 days post-stimulation.

### ELISA

IP-10, IFNγ and IL-13 ELISAs were performed using commercially available paired antibodies and recombinant cytokines (Becton Dickenson, Franklin Lakes, NJ). The limit of detection was 15.6 pg/ml for IP-10 and IFNγ, and 7.8 pg/ml for IL-13. IFNα was assayed via ELISA kit (PBL Interferon Source, Piscataway, NJ) according to the manufacturer's instructions; the limit of detection for IFNα was 9.7 pg/ml.

### Quantitative Real Time PCR

RNA was extracted using RNeasy Spin kit and RNA was reverse transcribed using Quantitect reverse transcription kit (Qiagen, Hilden, Germany), according to manufacturer's instructions. Gene expression was investigated by quantitative PCR (qPCR), using the ABI 7900 HT (Applied Biosystems, Foster City, CA USA) with Quantitect SYBR green (Qiagen). As the expression levels in unstimulated cultures were negligible and gene expression was induced by RV16, quantitation was achieved using standard curve analysis [25]. Ten-fold serial dilutions of PCR product standard were used to create standard curves for the gene of interest and the reference gene UBE2D2. UBE2D2 was initially identified as a stable reference gene in CD4+ cells [26] and subsequently assessed in-house to be stably expressed in total PBMC in the absence and presence of rhinovirus 16, using the method described by Silver et al [27]. The resultant ratio of gene of interest to reference gene were natural log transformed to normalise the data, allowing parametric statistical analysis to be performed. Table 1 shows the primers used to amplify cDNA. Copy numbers were determined by 10-fold serial dilution of PCR product standard and normalized to the reference gene UBE2D2 [26]. Data are expressed as a ratio of stimulated to control (unstimulated) samples.

**Table 1:** Primer sequences used for quantitative real time PCR amplification

Gene Name	Forward (5' - 3')	Reverse (3' - 5')
Ubiquitin containing enzyme E2D2 (UBE2D2)	ATGGCAGCATTTGTCTTGATATTCTAC	GGATTGGGATCACACAACAGA
Myxovirus protein A (MxA)	CTCGGCAACAGACTCTTCCAT	CATGAAGAACTGGATGATCAAAGG
2',5'-oligoadenylate synthetase (OAS)	AGAAATACCCCAGCCAAATCTCT	TGAGGAGCCACCCTTTACCA
Interleukin - 12 (IL-12)/p35	CCTTCACCACTCCCAAAACCT	CCTCCACTGTGCTGGTTTTATCT
Interleukin-23 (IL-23)/p19	TGGGACACATGGATCTAAGAGAAG	GAT CCT TTGCAAGCAGAACTGA

### Statistics

Statistical analysis was performed using SPSS 18 (IBM SPSS Inc., Chicago, IL, USA). After the data was log transformed, it was found to be normally distributed. Group comparisons were therefore made using paired two-tailed t-test, with p < 0.05 considered significant. Correlations between variables were made using Pearson's correlation. Raw data is presented as medians and interquartile range, while transformed data is presented as mean ± standard error of the mean (SEM).

## Results

Sixty three healthy volunteers were recruited for the study. The younger females were aged between 18.8 - 49.9 years, younger males were 19.0 - 47.6 years old, older females were 53.7 - 80.7 years old while older males were 51.9 - 81.5 years of age. Forty percent of subjects showed minor asymptomatic atopy (SPT mean wheal diameter ≥ 3 mm); none had symptomatic asthma or allergic rhinitis. The proportion of subjects with a positive SPT was similar in men and women.

**Figure 1:**
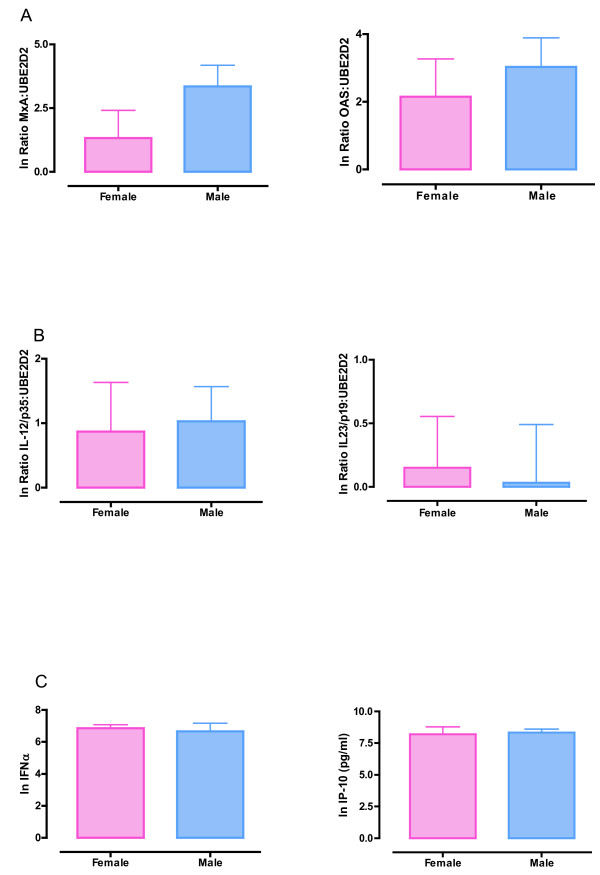
**Innate immune responses to rhinovirus**. PBMC from younger men and women (≤50 year old) were exposed to RV16 for 6 or 24 hours. RNA was extracted from cells at 6 hours post-infection, and reverse transcribed. Real-time PCR was performed and mRNA expression normalised to the control gene UBE2D2. (A) mRNA expression of MxA and OAS. (B) mRNA expression of IL-12/p35 and IL-23/p19. (C) Culture supernatants were collected at 24 hours post-infection and assayed for IFNα and IP-10 by ELISA. Data were natural log transformed and are presented as mean ± SEM.

The selected parameters of RV-induced innate immune function did not vary in relation to sex or age. Six hours after RV16 exposure, PBMC from men and women in the younger age group showed similar expression of the IFN stimulated genes myxovirus protein A (*MxA*, also known as *Mx1*) and 2',5'-oligoadenylate synthetase (*OAS*) (Figure 1a) and the Th1- and Th-17 polarising genes interleukin *IL-12p35 *and *IL-23p19*, as measured by qPCR (Figure 1b). Twenty four hours after PBMC were exposed to RV16, concentrations of IFNα (Figure 1c) and the chemokine IP-10 (Figure 1c; Table 2) in culture supernatants were similar in cells derived from men and women in the younger age bracket. Similarly, in PBMC from the older subjects the concentration of IP-10 in supernatant 24 hours after RV16 exposure was similar in men and women (Table 2; Additional File 1). As IP-10 is a type I IFN stimulated protein, and IP-10 synthesis was similar in older men and women, other molecules in the type I IFN pathway such as MxA, OAS and IFN-α were not assessed in the older cohort. Further details of cytokine production from both unstimulated and RV16 treated cultures can be found in Table 2.

**Table 2:** Median cytokine expression in unstimulated and rhinovirus 16 exposed cultures.

		Unstimulated		RV16 Treated	
		
Age	Cytokine (pg/ml)	Total cohort: Median	IQ range	Total cohort: Median	IQ range
Total Cohort	IP-10	180	77 - 499	5393	3481 - 6891 **
	IFNγ	0	0 - 17	1890	343 - 3915 **
	IL-13	0	0 - 44	47	0 - 87 **

		Female:	Male:	Female:	Male:
		Median and IQ range	Median and IQ range	Median and IQ range	Median and IQ range

≤50 years old	IP-10	150 (42 - 504)	258 (66 - 720)	4003 (2781 - 7252)	4571 (2927 - 5861)
	IFNγ	0 (0)	0 (0)	3423 (1766 - 4449)	969 (419 - 3525) *
	IL-13	43 (20 - 67)	23 (0 - 60)	70 (51 - 306)	36 (0 - 45) *

≥52 years old	IP-10	107 (75 - 370)	237 (119 - 526)	5917 (3932 - 6904)	5631 (3732 - 7153)
	IFNγ	0 (0)	0 (0)	1641 (205 - 4766)	1403 (158 - 3879)
	IL-13	0 (0 - 46)	0 (0)	11 (0 - 177)	33 (0 - 82)

PBMC were exposed to RV16 and culture supernatant was collected at 24 hours to measure IP-10, and at 5 days to measure IFNγ and IL-13. Median cytokine values, with interquartile (IQ) range for unstimulated and RV16 exposed are shown. Data was assessed for normality and natural log transformed before group comparisons were made using paired two-tailed t-test. Only comparisons attaining p < 0.05 were considered significant and only these p values are shown.

* Male vs. Female, RV16 treated: p < 0.01 ** Total cohort, unstimulated vs. RV16 treated: p < 0.001

We next examined the adaptive immune response in RV16 stimulated PBMC cultured for 5 days. RV16 exposure led to the synthesis of high concentrations of IFN-γ and much smaller concentrations of IL-13 (Table 2; median IFNγ = 1890 pg/ml and median IL-13 = 47.4 pg/ml, n = 63). In order to confirm that these responses were confined to pre-existing memory T-cells we investigated the effect of depletion of CD45R0+ cells in a subset of individuals (3 male and 2 female) using immuno-magnetic beads. The depletion of CD45R0+ cells led to a >98% reduction in IFNγ and IL-13 synthesis at day 5 after RV16 stimulation, whereas sham bead depletion had no effect (*data not shown*). It is therefore likely that these cytokine responses reflect a recall or memory immune response.

**Figure 2:**
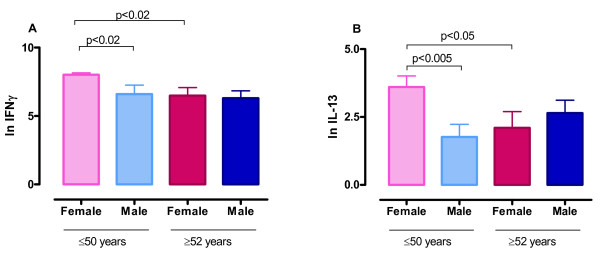
**Adaptive immune responses to rhinovirus**. PBMC from men and women were exposed to RV16 for 5 days. Culture supernatant was collected and assayed for IFNγ (A) and IL-13 (B) by ELISA. Data were natural log transformed and are shown as delta values (mean ± SEM) after subtracting control (unstimulated) values. Data are from 14 women and 10 men ≤50 years old, and from 18 women and 20 men ≥52 years old.

RV16-induced IFNγ production in the 5 day supernatant was significantly higher in PBMC from younger women than in cultures from age matched men (p < 0.02) or older women (≥52 years old; p < 0.02) (Figures 2a and Table 2). RV16 induced IL-13 production in the 5 day supernatant was also higher in PBMC from younger women than in age-matched men (Figure 2b and Table 2; p < 0.005) or older women (≥52 years old; p < 0.05). As assessed by Pearson's correlations, IFNγ and IL-13 concentrations in these day 5 cultures were inversely proportional to age in women, but not in men (Table 3). No association was observed between the presence or absence of atopy (positive SPT ≥ 3 mm) and IFNγ or IL-13 concentrations at day 5 (*data not shown*).

**Table 3:** Correlation between cytokine production and age.

Sex	Cytokine	Correlation with age
**Female**	**IFNγ**	**r = -0.40, p = 0.02**
Male	IFNγ	r = -0.22, p = 0.22
**Female**	**IL-13**	**r = -0.43, p = 0.01**
Male	IL-13	r = 0.06, p = 0.73

Correlations between variables were made using Pearson's correlation, with p < 0.05 considered to be statistically significant (shown in bold).

## Discussion

The key finding to emerge from this study is that particular aspects of cellular immunity to RV16 vary significantly with sex and age. In particular, PBMC from healthy pre-menopausal women make stronger adaptive immune responses to RV16 than men of the same age, with higher secretion of both the Th1 cytokine IFNγ and the Th2 cytokine IL-13. PBMC from pre-menopausal women also secrete more IFNγ and IL-13 than PBMC from post-menopausal women.

Because this sex difference is apparent in the younger subjects aged less than 50 years, but not in those aged 52 years or more, it is more likely to be linked to hormonal influences rather than genetic regulation. Sex hormones can all influence immune function, as reviewed elsewhere [17,18], though this has not been specifically studied in relation to RV. In a recent report, the capacity of IL-13 secreting T-cells to accumulate in culture was shown to be greater in healthy young women than in men [19]. Although Loza *et al *employed antigen-independent stimulation of T-cells, rather than a specific antigen, and post-menopausal women were not investigated [19], their findings are nonetheless consistent with the notion that immune function differs between women and men.

In contrast to adaptive immunity, our studies demonstrate that many aspects of the innate immune response to RV16 are similar in women and men. Expression of two IFN stimulated genes and two T-cell polarizing genes did not vary with sex, nor did concentrations of IFNα and IP-10 in culture supernatants of RV16 stimulated PBMC. A previous report has demonstrated that women have a higher capacity for IFNα synthesis than men [20], though in that study PBMC were stimulated with synthetic TLR7 ligands rather than with live virus.

We must acknowledge the limitations of the current study. Firstly, the evidence that the variations in RV16 induced IFNγ and IL-13 secretion is due to specific hormones is circumstantial at this stage. Oestrogen and/or progesterone are likely candidates, especially as IFNγ and IL-13 were inversely proportional to age in women, but not in men (Table 3). Hence, it will be important for future studies to examine this issue in more detail using appropriate hormone receptor agonists and antagonists. Secondly, information on the timing of blood collection in relation to the menstrual cycle in our female subjects was not recorded. While this could have influenced our findings to some degree, it is unlikely that the timing of blood collection explains the differences between the male and female responses shown in Figure 2 and Table 3. Future studies of cellular responses to RV that involve women in the child bearing years should consider this issue. Thirdly, it is possible that variations in the numbers of leukocytes between men and women might explain some of the variations in cytokine synthesis seen in this study. However, the consensus in the literature is that differences in differential leukocyte counts are not sufficient on their own to explain the variations in immune responses between men and women, and that sex hormones have a more profound effect on immunity [18].

It seems very likely that the RV16 induced cytokine synthesis observed at day 5 reflects a response by antigen experienced memory T-cells, especially as these responses were abolished by depletion of CD45R0+ cells from PBMC. We do not, however, know the extent to which the subjects in this study had previously been exposed to RV16 or to closely related RV strains. Although all subjects showed a strong IFNγ response to RV16, this does not necessarily mean that all responses were directed to RV16-specific T-cell epitopes. We may speculate that some memory T-cells were responding to epitopes shared across a number of RV strains. Currently, there is little information concerning T-cell epitopes to RV strains and this is a subject that clearly warrants further study.

It is important to reflect on whether the 'stronger' adaptive immune response in pre-menopausal women is truly associated with protection against RV. Greater capacity for IFNγ secretion would be predicted to reduce viral shedding [13,14], though it is possible this might be counteracted by higher IL-13 secretion. While Corne and colleagues showed no sex difference in the incidence of naturally acquired RV infection in a prospective study [28], there is little literature available on whether the severity of infection or duration of viral shedding in RV infection varies with sex and age. It is also possible that increased cytokine secretion by RV-specific T-cells in asthmatic women might actually contribute to the immunopathology of airway inflammation. These are clearly questions that require further investigation.

In conclusion, because adaptive immunity to RV varies in relation to sex and age, it is imperative that these variables are considered in the design of future studies of RV infection and cellular immune function in respiratory diseases. Adult asthma is more common in women than in men, so failure to properly match asthmatic and control groups for sex and age may produce spurious outcomes. Our findings are relevant to improved understanding of host defence against RV and will need to be considered in the development of new treatments and vaccines for RV infection.

## Conclusions

PBMC from healthy pre-menopausal women make stronger adaptive immune responses to RV16 than men of the same age and PBMC from post-menopausal women.

## Abbreviations

OAS: 2',5'-oligoadenylate synthetase; cDNA: Complementary deoxyribonucleic acid; ELISA: Enzyme linked immunosorbent assay; FCS: Fetal calf serum; IFN: Interferon; IP-10: Interferon gamma inducible protein 10; IL: Interleukin; mRNA: Messenger ribonucleic acid; MOI: Multiplicity of infection; MxA: Myxovirus protein A; PBMC: Peripheral blood mononuclear cells; RV: Rhinovirus; RV16: Rhinovirus serotype 16; RNA: Ribonucleic acid; SPT: Skin Prick Test; Th1: T Helper 1; Th2: T Helper 2; TLR7: Toll-like receptor 7

## Competing interests

The authors declare that they have no competing interests.

## Authors' contributions

MLC carried out the study and analysis. STY contributed to the study design and performed statistical analysis. ALP and JMD provided intellectual input in relation to data interpretation. JWU was the principal investigator of the study and was responsible for the study and protocol design, and interpretation of data. All authors assisted with writing the manuscript, and read and approved the final manuscript.

## Supplementary Material

Additional file 1 **Innate immune responses to rhinovirus in ≥52 year olds**. PBMC from older men and women (≥52 year old) were exposed to RV16 for 24 hours. Culture supernatants were collected and assayed for IP-10 by ELISA. Data were natural log transformed and are presented as mean ± SEM. Click here for file
