# Supplementary figures and images for: Adaptive immunity to rhinoviruses: sex and age matter
Source: Respir Res. 2010 Dec 31;11(1):184. doi: 10.1186/1465-9921-11-184 (PMC3024249; doi:10.1186/1465-9921-11-184)

# Additional File 1, Figure S1

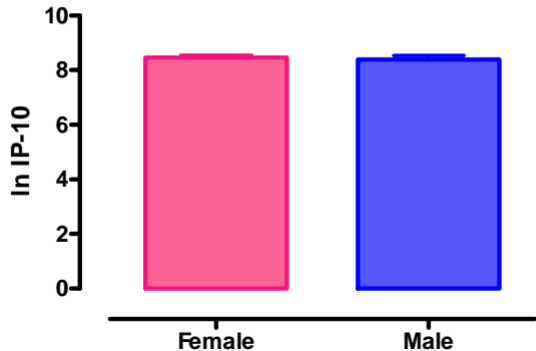

Supplement: Additional file 1 — Innate immune responses to rhinovirus in ≥52 year olds. PBMC from older men and women (≥52 year old) were exposed to RV16 for 24 hours. Culture supernatants were collected and assayed for IP-10 by ELISA. Data were natural log transformed and are presented as mean ± SEM. [file 1465-9921-11-184-S1.PDF]
